# Hypertrophic Cardiomyopathy in Children: Pathophysiology, Diagnosis, and Treatment of Non-sarcomeric Causes

**DOI:** 10.3389/fped.2021.632293

**Published:** 2021-02-25

**Authors:** Emanuele Monda, Marta Rubino, Michele Lioncino, Francesco Di Fraia, Roberta Pacileo, Federica Verrillo, Annapaola Cirillo, Martina Caiazza, Adelaide Fusco, Augusto Esposito, Fabio Fimiani, Giuseppe Palmiero, Giuseppe Pacileo, Paolo Calabrò, Maria Giovanna Russo, Giuseppe Limongelli

**Affiliations:** ^1^Department of Translational Medical Sciences, University of Campania “Luigi Vanvitelli”, Naples, Italy; ^2^Institute of Cardiovascular Sciences, University College of London and St. Bartholomew's Hospital, London, United Kingdom

**Keywords:** hypertrophic cardiomyopathy, etiology, children, diagnosis, treatment

## Abstract

Hypertrophic cardiomyopathy (HCM) is a myocardial disease characterized by left ventricular hypertrophy not solely explained by abnormal loading conditions. Despite its rare prevalence in pediatric age, HCM carries a relevant risk of mortality and morbidity in both infants and children. Pediatric HCM is a large heterogeneous group of disorders. Other than mutations in sarcomeric genes, which represent the most important cause of HCM in adults, childhood HCM includes a high prevalence of non-sarcomeric causes, including inherited errors of metabolism (i.e., glycogen storage diseases, lysosomal storage diseases, and fatty acid oxidation disorders), malformation syndromes, neuromuscular diseases, and mitochondrial disease, which globally represent up to 35% of children with HCM. The age of presentation and the underlying etiology significantly impact the prognosis of children with HCM. Moreover, in recent years, different targeted approaches for non-sarcomeric etiologies of HCM have emerged. Therefore, the etiological diagnosis is a fundamental step in designing specific management and therapy in these subjects. The present review aims to provide an overview of the non-sarcomeric causes of HCM in children, focusing on the pathophysiology, clinical features, diagnosis, and treatment of these rare disorders.

## Introduction

Hypertrophic cardiomyopathy (HCM) is a myocardial disease characterized by left ventricular (LV) hypertrophy not solely explained by abnormal loading conditions (1). HCM is a common genetic disorder in adults, with an estimated prevalence of 1:500 (1); on the contrary, it is rare in children but carries an important risk of morbidity and mortality (2). Compared with adult HCM, pediatric HCM is a more heterogeneous group of disorders. Other than mutations in sarcomeric genes, which represent the most important cause of HCM both in adults and in children (40–60% of cases) (1–7), the other causes of HCM include inherited errors of metabolism (i.e., glycogen storage diseases (GSDs), lysosomal storage diseases, and fatty acid oxidation disorders), neuromuscular diseases, malformation syndromes (i.e., RASopathies), and mitochondrial disease (Table 1), which globally represent up to 35% of children with HCM (2, 8). Sometimes, HCM is a feature of genetic syndromes associated with congenital heart disease (CHD), such as Noonan syndrome (NS), in which, for example, valvular pulmonary stenosis and HCM can be associated (3).

**Table 1:** Causes of hypertrophic cardiomyopathy in children.

**Sarcomeric**	
Malformation syndromes: RASopathies	Noonan syndrome Noonan syndrome with multiple lentigines Costello syndrome Cardiofaciocutaneous syndrome
Glycogen storage diseases	Pompe disease (glycogen storage disease type IIa) Danon disease (glycogen storage disease type IIb) Cori–Forbes disease (glycogen storage disease type III) PRKAG2 syndrome
Lysosomal storage diseases	Mucopolysaccharidoses
Mitochondrial disorders	
Fatty acid oxidation disorders	Very long-chain acyl-CoA dehydrogenase deficiency Multiple-acyl-CoA dehydrogenase Long-chain hydroxyacyl-CoA dehydrogenase Carnitine-acylcarnitine translocase Carnitine palmitoyltransferase II Carnitine-acylcarnitine translocase deficiency
Endocrine disorders	Primary hyperinsulinism Infant of a mother with diabetes mellitus Acromegaly

According to the 2020 American Heart Association/American College of Cardiology (AHA/ACC) guidelines, diagnosis of sarcomeric HCM in children requires an LV wall thickness more than 2 standard deviations from the predicted mean in children with a positive family history or a positive genetic test, and more than 2.5 in those without (9); however, specific *z* score thresholds have not been independently standardized.

The latest classification of cardiomyopathies in children of the AHA (10) has classified HCM in primary HCM, if a mutation in sarcomeric genes represents the cause of the disorder, and secondary HCM, if the disorder is associated with a non-sarcomeric cause; for the purpose of this document, we will refer to this classification.

**Figure 1:**
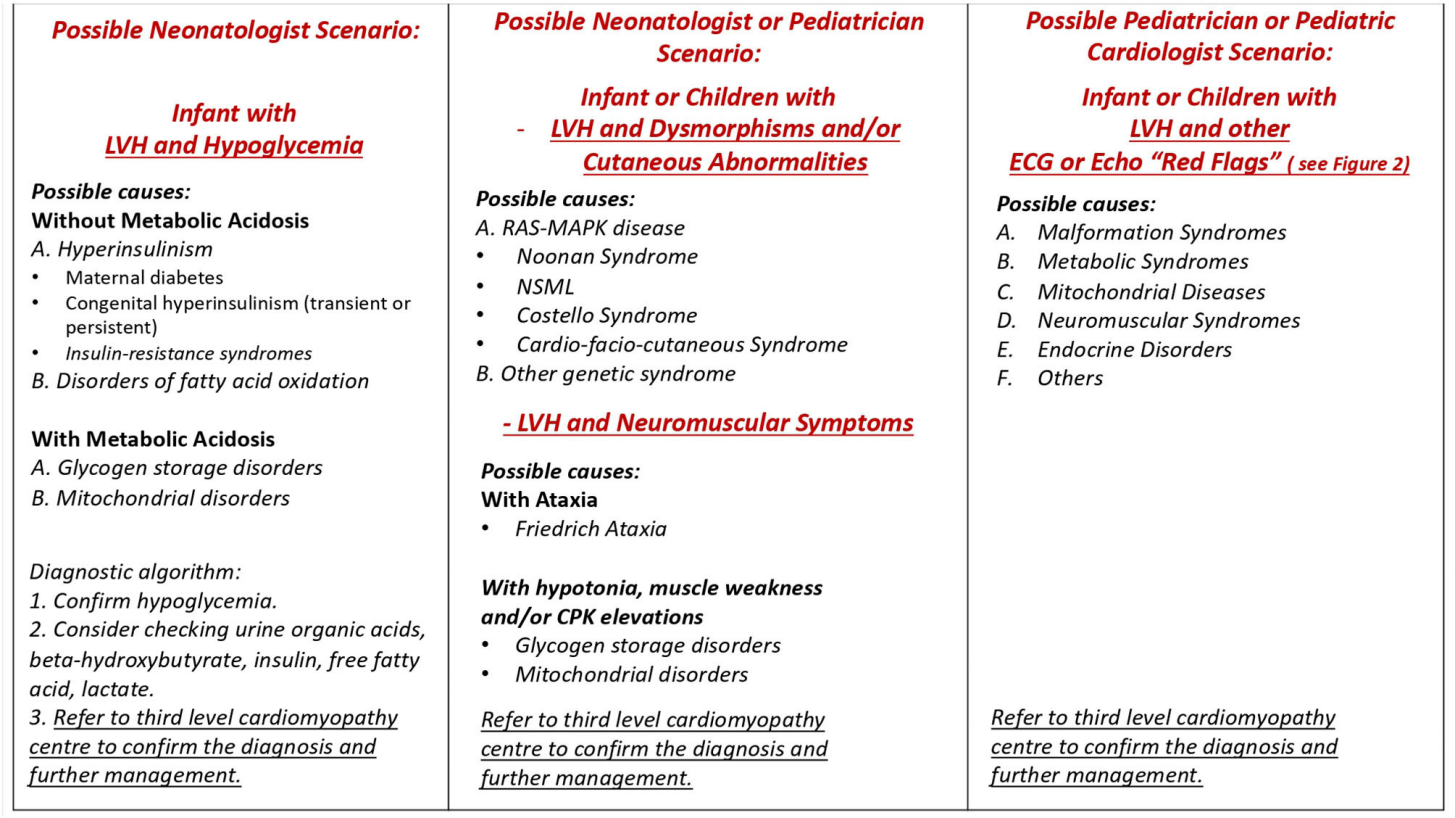
Possible scenarios in clinical practice. LVH, left ventricular hypertrophy; NSML, Noonan syndrome with multiple lentigines.

**Figure 2:**
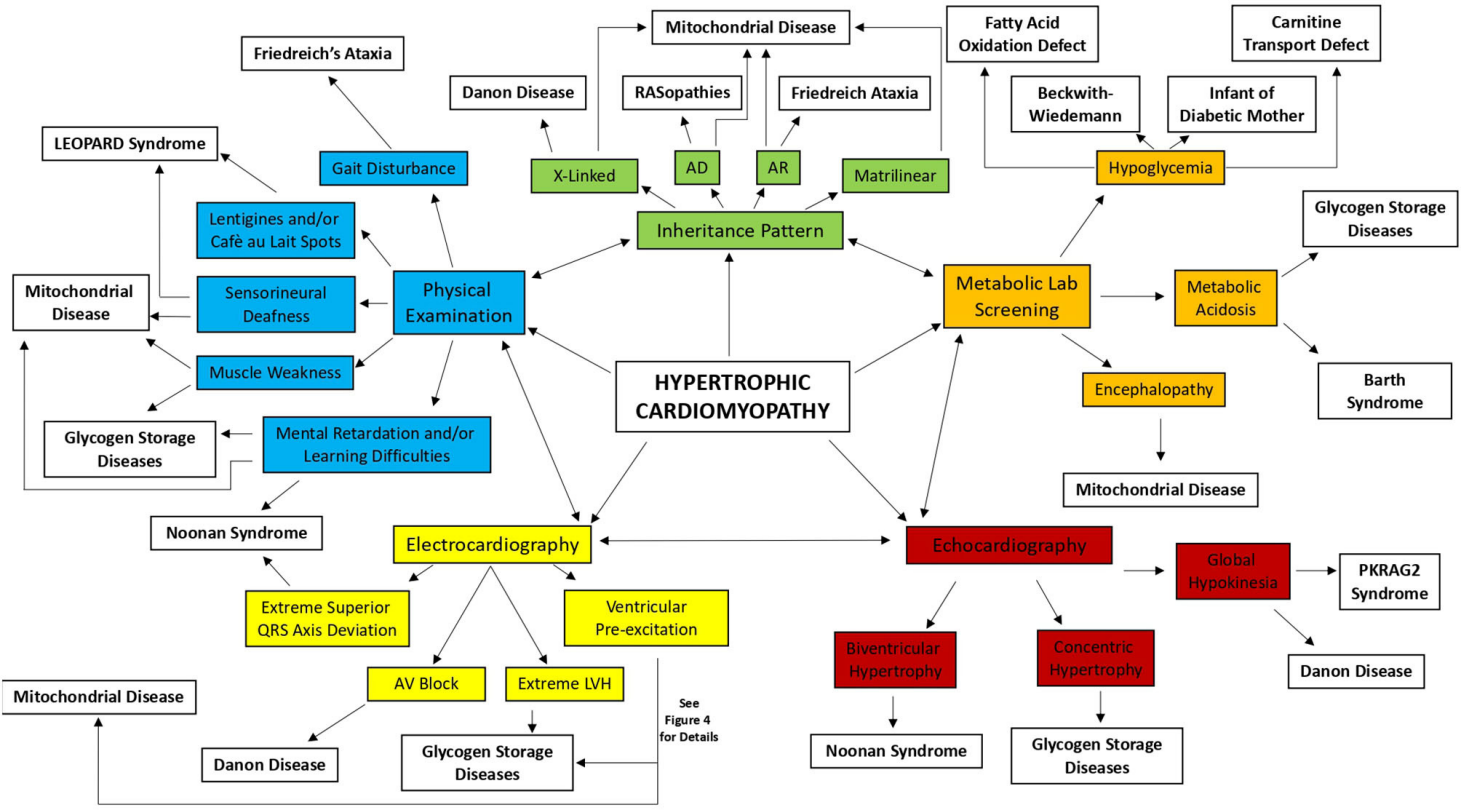
Diagnostic approach for the infant and young child with hypertrophic cardiomyopathy. AD, autosomal dominant; AR, autosomal recessive; AV, atrioventricular; LVH, left ventricular hypertrophy.

**Figure 3:**
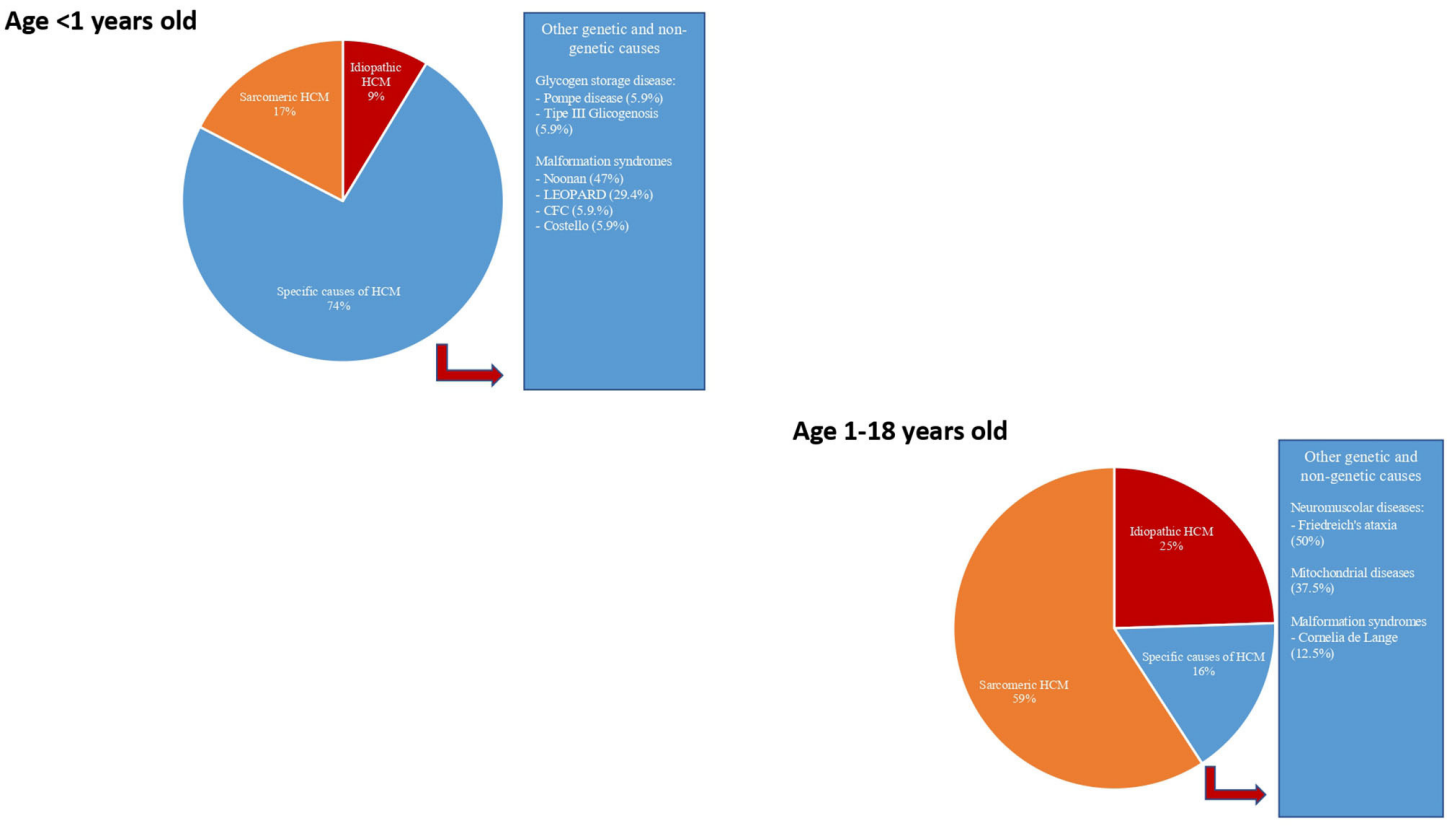
Etiology of hypertrophic cardiomyopathy in infants (age < 1 year) and in a young child (age 1–18 years). Modified from Limongelli et al. *Int J Cardiol* 2020 (3).

In non-sarcomeric HCM, although the increased LV wall thickness can simulate that of sarcomeric form, the pathophysiology, the natural history, and the treatment are different. Thus, in subjects who meet the threshold for HCM diagnosis, causal predisposition or addition phenotypic characteristics need to be evaluated to identify the underlying cause (1, 3, 10, 11). To early identify the etiology of HCM, several diagnostic markers (obtained by pedigree analysis, physical examination, electrocardiography, echocardiography, and laboratory tests), the so-called “red flags,” have been recommended to guide specialized diagnostic testing, including genetic analysis (1, 3, 10, 11) (Figures 1, 2). The age of presentation can be considered a “red flag” for specific causes of HCM and also has a prognostic role. For example, HCM presenting before 1 year of age shows a worse prognosis (2) and is principally caused by inherited errors of metabolism or malformation syndrome (e.g., RASopathies) (Figure 3). On the contrary, mutations in sarcomeric genes represent the most important causes of HCM outside infancy, with a better prognosis compared with non-sarcomeric forms of HCM (2, 12, 13).

In recent years, several targeted approaches for specific etiologies of HCM have emerged (14, 15). Thus, the etiological diagnosis of HCM in children is a fundamental step in designing specific management and therapy in these subjects. The present review aims to provide an overview of the specific causes of HCM in children, focusing on the pathophysiology, diagnosis, and treatment of these rare disorders.

## Malformation Syndromes: RASopathies

### Introduction

RASopathies are a group of genetic disorders caused by mutations in RAS-MAPK cascade, which constitute, taken together, one of the largest groups of malformation syndromes, with a prevalence of 1:1,000–2,500 children (16–18). They include NS, NS with multiple lentigines (NSML; formerly known as LEOPARD syndrome), Costello syndrome (CS), cardiofaciocutaneous syndrome (CFC), neurofibromatosis type 1 (NF1), and Legius syndrome (LS).

Besides cardiac involvement, these diseases show a common clinical pattern whose major features are craniofacial dysmorphology, hypotonia, neurocognitive impairment, short stature, predisposition to various pediatric cancers, and cutaneous, muscular, and ocular abnormalities (19–21).

### Clinical Presentation and Diagnostic Considerations

#### Noonan Syndrome

NS [Online Mendelian Inheritance in Man (OMIM) code #163950] is a developmental multisystemic disorder transmitted with an autosomal dominant pattern (17), and it is the second most common syndromic cause of CHD after trisomy 21 (22). Patients affected by NS are characterized by specific craniofacial features, including broad forehead, down slanting palpebral fissures, hypertelorism, and low-set ears; almost 10% of them have neurosensorial deafness or auditory deficits in the low-frequency range (17). Short stature is a common manifestation of the syndrome as the puberal grow spurt is usually attenuated (23). Up to 80% of male patients have unilateral or bilateral cryptorchidism (24). Among dermatological abnormalities, hypo- or hyperpigmentation can occur, including cafè-au-lait spots, keratosis pilaris, or multiple pigmented nevi (25). In most individuals, intelligence is within the normal range; nonetheless, Roelofs et al. showed that almost 60% of the individuals have a significant difference between verbal and non-verbal intelligence quotient (26). Several malignancies have been reported in patients with NS, particularly juvenile myelomonocytic leukemia, acute myelogenous leukemia, and embryonal rhabdomyosarcoma (27).

The first NS pathogenetic gene was described in 2001 by Tartaglia et al. and identified as the tyrosine phosphatase *PTPN11*, whose missense loss-of-function mutations cause overactivation of the RAS-MAPK cascade (28). Subsequent studies showed that *PTPN11* accounts for almost 50% of the cases (29) (Table 2). *SOS1* mutations represent the second most common cause of NS, accounting for almost 15% of the patients (30). A rare subset of NS patients who show a particular phenotype of loose anagen hair (also known with the eponym of Mazzanti syndrome) is caused by a single mutation is *SHOC2*, a scaffold protein linking Ras to *RAF1* (31). Later studies identified other genes underlying NS, namely, *KRAS* (32), *RAF1* (33), *MAP2K1* (34), *BRAF* (35), *NRAS* (36), and *CBL* (37).

**Table 2:** Clinical manifestations, mutated genes, and classical heart defects with their relative prevalence among different specific causes of pediatric HCM.

**Specific cause**	**Gene**	**Clinical features**
**RASopathies**		
Noonan syndrome	*PTPN11* *SOS1* *RAF1* *RIT1* *SHOC2* *NRAS* *CBL*	PVS (65%), HCM (20–23%), atrioventricular septal defects (10–18%), ASD (10%), VSD (8.5%), PDA (6%), mitral valve abnormalities (2.5%), tetralogy of Fallot (<1%), aortic coarctation (<1%). PVS (70%), HCM (18%), ASD (14.5%), mitral valve abnormalities (3.6%). HCM (65%), PVS (21%), ASD (19.6%), mitral valve abnormalities (13%), tetralogy of Fallot (2.8%), aortic coarctation (1.8%). PVS (74%), HCM (36%), ASD (30%), PDA (7.4%), mitral valve abnormalities (13%), biventricular obstruction (<1%). PVS (32%), ASD (32%), HCM (29.7%), mitral valve abnormalities (27%), VSD (12.7%). HCM (39.3%), PVS (14.3%). PVS (50%), HCM (10%), ASD (10%), mitral valve abnormalities (10%).
Noonan syndrome with multiple lentigines	*PTPN11* *RAF1* *BRAF*	HCM (60–85%), biventricular hypertrophy (46%), LVOTO (40%), ventricular tachycardia, conduction abnormalities, mitral valve abnormalities (26–42%), PVS (21%), ASD (6%), atrioventricular septal defects, coronary artery abnormalities. HCM (100%), mitral valve abnormalities (100%), PVS (40%), high risk of SCD. HCM (60–85%).
Costello syndrome	*HRAS*	HCM (65%), atrial tachycardia (56%), PVS.
Cardiofaciocutaneous syndrome	*BRAF1* *MAP2K1–MAP2K2:* *KRAS*	PVS (45%), HCM (40%), ASD (30%), VSD (7.6%). tetralogy of Fallot (7%). PVS (65–100%), HCM (15–40%), septal defects (15%). PVS (40%), mitral valve abnormalities (26%), HCM (20%), ASD (20%), VSD (13%).
**Glycogen storage disorders**		
Pompe disease	*GAA*	LVH, short PR interval, hypertension, idiopathic stroke, cerebral artery aneurysms.
Danon disease	*LAMP2*	Short PR interval (75%), severe concentric LVH, conduction abnormalities.
Cori–Forbes disease	*AGL*	Concentric LVH (40%), biventricular hypertrophy (15%), HF, isolated septal hypertrophy.
PRKAG2 syndrome	*PRKAG2*	LVH, AV block (45–50%), SVT (38%), sick sinus disease (50%), SCD (10%), syncope.
**Lysosomal storage disorders**		
Hurler syndrome (MPS type 1)	*IDUA*	LVH, thickening of valve leaflets or papillary muscles, shortening of subvalvular apparatus, systemic hypertension, dilated cardiomyopathy with reduced EF, pulmonary hypertension, coronary artery disease, AV block.
Hunter syndrome (MPS type 2)	*IDS*	LVH, thickening of valve leaflets or papillary muscles, shortening of subvalvular apparatus, systemic hypertension, dilated cardiomyopathy with reduced EF, pulmonary hypertension, coronary artery disease, AV block.
Maroteaux–Lamy syndrome (MPS type 6)	*ARSB*	LVH, thickening of valve leaflets or papillary muscles, shortening of subvalvular apparatus, systemic hypertension, dilated cardiomyopathy with reduced EF, pulmonary hypertension, coronary artery disease, AV block.
**Mitochondrial disorders**		
MELAS syndrome	Mitochondrial genes	HCM (75%), dilated cardiomyopathy (12.5%), pulmonary hypertension (12.5%), stroke-like episodes.
MERRF syndrome	Mitochondrial genes	WPW (45–22%), dilated cardiomyopathy (22%), HF (11%).
CoQ10 deficiency	*COQ4*	HCM, bradycardia, HF.
Barth syndrome	*TAX*	Left ventricular non compaction, dilated cardiomyopathy, HCM, endocardial fibroelastosis, ventricular arrhythmias, SCD.
Friedrich's ataxia	*FTX*	HCM (75%), dilated cardiomyopathy, granular sparkling (rare), bundle branch block (15%).

*ASD, atrial septal defect; HCM, hypertrophic cardiomyopathy; HF, heart failure; LVH, left ventricular hypertrophy; LVOTO, left ventricular outflow tract obstruction; PDA, patent ductus arteriosus; PVS, pulmonary valve stenosis; SCD, sudden cardiac death; VSD, ventricular septal defect; WPW, Wolff–Parkinson–White syndrome*.

### Noonan Syndrome With Multiple Lentigines

Among RASopathies, NSML, also known as LEOPARD syndrome (OMIM code #151100), is similar to NS, but it is characterized by multiple lentigines, sensorineural deafness, ocular hypertelorism, abnormal genitalia, and a higher prevalence of HCM and conduction abnormalities (38). Genetic analysis showed that NSML, similar to NS, is caused by different mutations in *PTPN11* (39). Rarely, mutations in *RAF1* and *BRAF*, which are more common in CFC, have been identified in NSML (39, 40).

### Costello Syndrome

CS (OMIM code #218040) is a genetic syndrome with many overlapping features with NS (19). Differential diagnosis with NS is seldom feasible during infancy and becomes possible only in adult life (41). Both syndromes can present HCM at very infantile onset, but infants with CS have a lesser prevalence of pulmonary stenosis or complex CHD (40–42). Phenotypic features may be apparent in intrauterine life, with polyhydramnios *in utero*, prematurity, and increased birth weight. Children with CS have coarse facial features, with a prominent forehead, epicanthal folds, and depressed nasal bridge. The skin is soft and redundant with palmar and plantar creases. Almost 72% of CS patients show evidence of cutaneous papilloma, localized especially at the nose. These neoplasms are not common in other RASopathies and could offer a clue for differential diagnosis (19, 42). CS is caused by heterozygous activating mutations in *HRAS*. More than 80% of CS patients have a p.G12S substitution, which causes reduction of the GTPase activity of HRAS, with subsequent overactivation of the MAPK pathway (43).

### Cardiofaciocutaneous Syndrome

Among the rarest RASopathies, CFC (OMIM code #115150) shows many overlapping features with NS and CS, including the typical facies, neurocognitive delay, hypotony, and learning disability (20). Differential diagnosis includes the presence of curly hair with sparse eyebrows and eyelashes (44), and neurological and ocular abnormalities like strabismus and nystagmus (45). Unlike CS, the genetic substrate of CFC is heterogeneous, with mutations in *BRAF1* (34), *MAP2K1, MAP2K2* (46), and *KRAS* accounting for 80% of the cases.

### Neurofibromatosis Type 1 and Legius Syndrome

RASopathies also include NF1 (OMIM code #162200) and LS (OMIM code #611431), caused, respectively, by inactivating mutations of *NF1*, which encode for a GTPase activating protein, and SPRED1, which is a negative mediator of RAS-mediated RAF 1 activation. However, HCM is not commonly associated with NF1 and LS.

### Hypertrophic Cardiomyopathy in RASopathies

With the exclusion of CHD, HCM is the most common cardiovascular abnormality observed in RASopathies (47, 48). HCM in RASopathies is characterized by a higher grade of ventricular hypertrophy, an increased prevalence, and a more severe pattern of LV outflow tract obstruction (LVOTO) than non-syndromic forms (49). Biventricular involvement is often described, because of the high prevalence of right ventricular hypertrophy (49). Obstructive forms of HCM are not solely explained by the systolic anterior motion of the mitral valve (MV), but other factors, such as accessory fibrous connective tissue causing subaortic stenosis, displacement of the papillary muscles, and anomalous insertion of the MV, have been described (47). The length of MV leaflets and chordal anatomic relationships are different compared with MV in non-syndromic subjects with HCM (50). To date, MV anomalies are a marker of complexity in HCM and have been associated with a high risk of reintervention and death (51). Myocardial ischemia is a common finding in HCM, reflecting the imbalance between oxygen supply and demand, and it is a major clinical issue in young adolescents with RASopathies (47). Coronary arteries abnormalities have been described in up to 30% of patients affected by RASopathies, and they could contribute to myocardial ischemia (52).

Moreover, cardiac arrhythmias are a major determinant of clinical prognosis in children affected by RASopathies and HCM (42). Supraventricular and ventricular ectopy have been described, and in severe cases, the occurrence of sustained ventricular tachycardia has been reported.

The initial evaluation of a patient affected by HCM should include systematic research for clinical clues or “red flags,” obtained by pedigree analysis, clinical examination, ECG, imaging, and biochemical tests. In particular, the diagnosis of RASopathy may be suggested by facial dysmorphism, lentigines, sensorineural deafness, pulmonary stenosis, or biventricular hypertrophy (3, 53).

### Hypertrophic Cardiomyopathy in Noonan Syndrome With Multiple Lentigines

HCM is diagnosed in almost 80% of patients affected by NSML, and it is associated with early-onset presentation and worse clinical outcome, with clinical evidence of LVOTO in up to 40% of the cases (38, 47, 54). Although HCM can be congenital in NSML, it is mostly observed in second infancy, where it precedes the appearance of multiple lentigines. Biventricular hypertrophy can be found in 46% of patients affected by NSML, in association with typical electrocardiographic findings, as a superiorly oriented mean QRS axis, q waves, prolonged QTc, and/or repolarization abnormalities (38). Clinically relevant genotype–phenotype correlations have been described: patients without PTPN11 mutations showed a higher prevalence of family history of sudden death (*p* = 0.007) and non-sustained ventricular tachycardias (*p* = 0.05). Of note, mutations of the exon 13 of the PTPN11 gene were associated with severe obstructive HCM (48).

Other cardiac manifestations in NSML include pulmonary valve stenosis, found in almost 23% of the affected individuals, and MV prolapse, clefting, or other morphological abnormalities, which are present in up to 42% of patients. Rarely, NSML has been associated with atrioventricular septal defects, LV non-compaction, and coronary arteries abnormalities (21).

### Hypertrophic Cardiomyopathy in Costello Syndrome

Approximately 65% of patients affected by CS have HCM, and 40% of them show evidence of CHD (42). Most patients have subaortic septal hypertrophy; however, other patterns such as concentric LV hypertrophy, mild septal thickening, or biventricular involvement have been described (42). In a cohort of 126 patients affected by CS, the mean age at diagnosis was 4.6 years, and no fetal diagnosis of HCM was made. The clinical course was progressive in 38% of the patients and stable in 30% of the patients, and interestingly, almost 11% of the patients showed regression of cardiac hypertrophy.

Although atrial arrhythmias are common among patients with RASopathies, their prevalence seems to be higher in CS, where they can be diagnosed in up to 56% of the patients. Thus, the identification of focal atrial tachycardia could represent a diagnostic clue and, in combination with specific clinical features, should trigger the suspicion of an underlying CS (19, 32, 43). On the other hand, in patients with other RASopathies, multifocal atrial tachycardia (MAT) seems to be associated specifically with RAF1 mutations (42).

### Hypertrophic Cardiomyopathy in Cardiofaciocutaneous Syndrome

HCM is diagnosed in almost 40% of individuals affected by CFC, although the most common cardiac abnormality is pulmonary valve stenosis, diagnosed in almost 45% of the patients (20). There are lacking data to compare HCM in CS to NS and CFC, although case reports suggest that severe subaortic obstruction requiring surgery is more frequent in CS than in NS and CFC (55).

### Hypertrophic Cardiomyopathy in Noonan Syndrome

A lower prevalence of HCM has been described for NS: although more than 80% of the patients show cardiac abnormalities, the prevalence of HCM in NS has been estimated to be 20–23% (17). Prevalence of HCM is slightly higher in a subset of Noonan patients carrying the variant p.Ser2Gly in SHOC2 gene, also known as NS with loose anagen hair (31). NS-associated HCM occurs early in childhood, with a median age at diagnosis of 6 months (56). The distribution of LV hypertrophy is similar to idiopathic variants. Asymmetric septal hypertrophy is the most common pattern, described in 75.6% of Noonan patients. Noteworthy, apical cardiomyopathy is seldom described in NS (57). Electrocardiograms may show several abnormalities, also in the absence of structural abnormalities (56). The magnitude of LV hypertrophy was significantly higher in those with NS or NSML than non-syndromic forms [8.9 as opposed to 6.4 (*p* = 0.03)]. Significant obstruction of LVOT is more common in NS or NSML than in idiopathic HCM (53 vs. 15%, *p* = 0.02). Dilatation of the coronary arteries has been described in 29% of the affected patients (49). Patients affected by NS and HCM also seemed to present a higher risk of dilated cardiomyopathy in adult life (21). The prognosis of the patients affected by NS and HCM is influenced by the high prevalence of pulmonary valve stenosis (described in almost 65% of the patients), and the coexistence of complex CHD as atrioventricular septal defects (10%), atrial septal defect (10%), and rarely tetralogy of Fallot, patent ductus arteriosus, and left-sided obstructions resulting in MV abnormalities (17, 21). Vascular abnormalities are also described, mainly aortic dissection, aortic root dilatation, and aneurysms of the sinuses of Valsalva (17).

### Genotype–Phenotype Correlation in RASopathies: The Key to a Target Therapy?

Many clinically relevant genotype–phenotype correlations have been described in RASopathies (Table 2). Among patients with NS, pulmonary stenosis was more common in *PTPN11* and *SOS1* mutation patients, whereas HCM without pulmonary stenosis was more prevalent in carriers of *RAF1* mutations, where the prevalence of HCM is up to 65% (16, 40, 58). It has been suggested that HCM in RASopathies could be caused by increased activation through the RAS-MAPK cascade, causing cardiomyocyte hypertrophy and myocardial fiber disarray (21). Consequently, treatment with Mek1 inhibitors seemed to rescue the cardiac phenotype in mouse Raf1-mutated models (59). Trametinib, a highly selective Mek1/2 inhibitor that seemed to reverse hypertrophic phenotype within 4 months after initiation of treatment, is preceded by a favorable clinical response in a single patient with *RIT1*-associated HCM (60). On the other hand, specific missense mutations of *PTPN11* associated with NSML seem to cause increased activity through the mTOR–PI3K–AKT signaling pathway. Treatment of the *PTPN11* (Y279C) mice with rapamycin, an inhibitor of mTOR–PI3K–AKT pathway, normalized HCM (61). Recently, everolimus has been used to prevent acute decompensation of heart failure in severe HCM in patients with NSML, even though no reversal of HCM was observed (62) (Table 3).

**Table 3:** Advantages and disadvantages of the available treatments for Pompe disease.

**HCM specific cause**	**Specific therapies and future perspective**
RASopathies	*No specific treatment currently available* *MEK inhibitors (future perspective)* In mouse models of Noonan syndrome, the administration of MEK inhibitors during 4–10 weeks of life could prevent the development of cardiomyopathy. Data from a clinical report showed that MEK inhibitors can induce the regression of cardiac hypertrophy within 4 months of treatment. *mTOR inhibitors (future perspective)* In mouse models with PTPN11 mutation Y279C, the administration of rapamycin, an inhibitor of mTOR–PI3K–AKT pathway, induced regression of HCM.
Pompe disease	*Enzyme replacement therapy* Advantages: reduction of cardiac mass and reverse remodeling; long-term efficacy achieved in a subgroup of patients. Disadvantages: high doses required; highly dependent on level of M6PR; does not cross the BBB; possible adverse reactions: ventricular ectopy; transient fall of the left ventricular ejection fraction; immune-mediated reaction. *Gene therapy (future perspective)* Advantages: improved clearance of glycogen in the muscles; enhanced respiratory and cardiac performance; potential for expressing GAA directly in target tissues. Disadvantages: high vector doses needed; transgene immunogenicity
Danon disease	*No specific treatment currently available* *Gene therapy (future perspective)* In mouse models of Danon disease, LAMP2B gene transfer improves metabolic and physiologic function.
PRKAG2 syndrome	*No specific treatment currently available*
Cori–Forbes disease	*No specific treatment currently available* *Gene therapy (future perspective)* *In mouse models of Cori*–*Forbes disease, GDE gene transfer blocked glycogen accumulation in cardiac and skeletal muscles and improved the muscle functions*.
Mucopolysaccharidoses	*Enzyme replacement therapy* Advantages: effective on visceral organs; easy to administer. Disadvantages: has limited impact on poorly vascularized tissues; does not cross the BBB; requires continuous administration; can cause IgG antidrug antibodies formation; can cause infusion-related reactions. *Hematopoietic stem cell transplantation* Advantages: it is a permanent therapy; increases the long-term survival; can cause the interruption of the disease progression; can cross the BBB. Disadvantages: does not prevent valve dysfunction; can cause GVHD; can cause graft rejection. *Gene therapy (future perspective)* Advantages: it is a permanent therapy; potentially able to cross the BBB. Disadvantages: can cause off-target gene effects; the long-term effects are unknown. *Chaperone therapy (future perspective)* Advantages: can cross the BBB; it is not immunogenic. Disadvantages: can cause off-target adverse effects; it is not approved for all the MPSs type.
Mitochondrial disorders	*No specific treatment currently available, except for:* *CoQ_10_ deficiency:* CoQ_10_ supplementation. *Thiamine-responsive disorders:* thiamine supplementation. In mouse models of Barth syndrome, gene therapy rescued neonatal demise, prevented cardiac dysfunction, and reversed established heart disease. Several treatments are currently on investigations, such as MitoQ antioxidant, MTP-131 peptide (which reduce the ROS release and improving ATP synthesis), inhibitors of mPTP, mitochondrial transplantation, etc. (63)

*BBB, brain–blood barrier; ERT, enzyme replacement therapy; GVHD, graft vs. host disease; M6PR, mannose-6-phosphate receptor; MPSs, mucopolysaccharidoses*.

### Prognosis and Risk of Sudden Cardiac Death

In the subgroup of patients with RASopathies, HCM is a major determinant of the clinical prognosis. In a cohort study, children with NS presented a higher prevalence of congestive heart failure (CHF) (24 vs. 9%) compared with those with idiopathic HCM, and a significant early mortality rate (22% at 1 year) (64). Low cardiac output, significant diastolic dysfunction, and a higher number of interventions have been reported in patients who died for cardiac causes (51, 64).

The stratification of risk for sickle cell disease (SCD) among patients with RASopathies and HCM is not completely understood, and data have been extrapolated from larger studies including pediatric patients affected by sarcomeric HCM (65–67). Recently, an international multicentric observational cohort study including 572 children with HCM has validated age at diagnosis, history of recent unexplained syncope within 6 months before the diagnosis, documented non-sustained VT (defined as ≥3 beats at ≥120 bpm on ambulatory ECG), interventricular septal diameter (IVSD) *z* score, LV posterior wall diameter (LVPWD) *z* score, left atrial (LA) diameter *z* score, and peak resting LVOT gradient on echocardiography as risk factors for SCD in pediatric HCM. Nevertheless, patients with RASopathies were excluded from the analysis (68).

Specific etiologies, as the diagnosis of NSML and, in particular, an arrhythmogenic phenotype as the absence of PTPN11 mutation in NSML, might provide additional risk, but the available evidence is not conclusive to provide a prognostic stratification for SCD in children with RASopathies (48).

### Treatment

General management of HCM in RASopathies is based on current clinical practice guidelines. Medical therapy, mainly based on beta-blockers, can be used to relieve symptoms and the degree of obstruction (1).

Surgical myectomy is feasible in NS and should be considered in patients who remain symptomatic despite maximal medical therapy (69). Orthotopic heart transplantation is rarely performed, and long-term follow-up studies are needed to assess prognostic implications of heart transplantation in these patients (70). However, it may be considered in those with advanced heart failure, or intractable ventricular arrhythmias, or severe diastolic dysfunction.

## Glycogen Storage Diseases

GSDs represent an important cause of HCM in children and are characterized by the formation of glycogen-filled vacuoles in cardiomyocytes (71). Different GSDs are known to be associated with HCM (1, 72, 73); in particular, the most important are represented by Pompe disease (or GSD type IIa), Danon disease (or GSD type IIb), Cori–Forbes disease (or GSD type III), and PRKAG2 syndrome.

### Pompe Disease (Glycogen Storage Disease Type IIa)

#### Introduction

Pompe disease (OMIM code #232300) is a rare and progressive metabolic disorder caused by the partial or complete deficiency of the acid alpha-glucosidase enzyme (GAA). This condition leads to a pathological accumulation of glycogen in several organs, in particular in the nervous system and muscles, including the heart (74).

It is inherited with an autosomal recessive pattern and is the result of homozygotic mutations in *GAA* (75), which encodes for an enzyme that is responsible for lysosomal glycogen hydrolyzation into glucose. The glycogen accumulation and the lysosomal membrane rupture in the muscle tissue lead to a massive leakage of lytic enzymes responsible for muscle damage (76, 77).

### Clinical Presentation and Diagnostic Consideration

Pompe disease is classified in infantile form and late onset, when it presents before and after the 1st year of life, respectively.

The infantile form is generally classified into a classic Pompe disease, which generally presents with a rapidly progressive course with severe cardiomegaly, hepatomegaly, weakness, hypotonia, respiratory distress, infections, and feeding difficulties; and a non-classic Pompe disease, which represents a variant form with slower disease progression and mild or absent cardiomyopathy (78–80). Cardiac involvement of the infantile form of Pompe disease represents the dominant feature of the disease and the major determinant of mortality. Thus, in the infantile form of Pompe disease, chest radiography often shows cardiomegaly, while ECG shows a short PR interval and hypertrophy signs (76). Echocardiography often shows LV hypertrophy with or without LVOTO, mimicking HCM. In particular, cardiac involvement presents in the 1st months of life with severe ventricular hypertrophy (ranging from +2 to +10 or higher *z* score for LV mass), most notably in the posterior wall and the interventricular septum, which can appear also in intra-uterine life. The LV ejection fraction, initially preserved, tend to fall in the first 5 months moving toward dilated cardiomyopathy with end-stage heart failure in the 1st year of age (81).

The late-onset form includes childhood, juvenile, and adult variants characterized by a slow course and a predominant skeletal muscle involvement (76). Other features of the late-onset form are represented by hypertension, due to loss of aortic compliance, and idiopathic stroke. Preexcitation syndrome, LV hypertrophy, and ascending aorta dilation represent possible cardiac features of late-onset Pompe disease. Respiratory failure is a possible lethal complication caused by the involvement of the respiratory muscle (82).

The diagnosis of Pompe disease is challenging given its heterogeneous presentation. Dry blood spot test is a valid screening method for patients with suspicion of Pompe disease, but the gold standard diagnostic test is the measurement of GAA enzyme activity in skin fibroblasts (74, 76). Indeed, the diagnosis is generally performed demonstrating a significant reduction in GAA enzyme activity and identifying the disease-causing mutations of the GAA gene.

### Prognosis and Risk of Sudden Cardiac Death

As stated before, cardiovascular involvement is the major determinant of prognosis in patients with the infantile form of Pompe disease, with the possible evolution to refractory heart failure and death in the 1st year of age (76, 81). On the contrary, in the late-onset form, the prognosis is mainly dependent upon the age of onset with a slower disease progression in those patients manifesting later the disease. In this subgroup, the prognosis is dependent upon the extent of respiratory muscle involvement (82). Sudden cardiac death is extremely rare in patients with Pompe disease.

### Treatment

Actually, the standard of care in patients with Pompe disease is represented by enzyme replacement therapy (ERT), based on the discovery of receptor-mediated uptake of lysosomal enzymes related to mannose-6-phosphate receptor (M6PR) (Table 3). The main goal of ERT is to slow down, stabilize, and reverse disease progression. Among the benefits of ERT, there is the reduction of cardiac mass, reverse remodeling, and improvement of cardiac function. The efficacy of this treatment has led to a reduction in the use of inotropes, diuretics, and vasodilators which can exacerbate the LVOTO (76). Recombinant GAA (rhGAA) doses used in patients with Pompe disease are markedly high compared with those used in other lysosomal storage diseases, probably because of the elevated liver absorption (more than 85%), limiting the muscle uptake. Possible side effects are rare and include a transient fall in LV ejection fraction (mostly in the first 12–24 weeks of therapy), ventricular ectopy, and immune-mediated reactions (83). Thus, to improve enzyme bioavailability in tissues, two strategies have been developed: the use of new rhGAA with high affinity for M6PR and the use of pharmacological adjuvants such as beta2agonists (e.g., clenbuterol and albuterol) or chaperones (e.g., glucose analogs duvoglustat and miglustat) (84).

Given the monogenic origin of Pompe disease, it represents an ideal target for gene therapy. In particular, recent progress has been made in the field of gene therapy mediated by adeno-associated virus vectors (AVV), and several preclinical studies demonstrated the efficacy of this treatment based on the improved clearance of glycogen in the muscle and the enhanced respiratory and cardiac performance. However, the limitations are primarily the high vector doses needed and the transgene immunogenicity caused by the increased muscle-specific GAA expression (84–86).

### Danon Disease (Glycogen Storage Disease Type IIb)

#### Introduction

Danon disease (OMIM code #300257) is a rare X-linked disorder with prominent effects on skeletal and cardiac muscle (87), caused by mutations in the *LAMP2* (88). The real prevalence of this condition is unclear; however, a disease-causing mutation in *LAMP2* has been found in up to 1% of HCM patients (72). LAMP2 is a glycosylated protein that is important in the prevention of the lysosome degradation from its acid hydrolases (89, 90). The impairment of LAMP2 protein function due to gene mutation leads to the accumulation of autophagic material and glycogen (91).

### Clinical Presentation and Diagnostic Consideration

Given the X-linked inheritance pattern, the clinical manifestation and the age of presentation are quite different in the two sexes. In males, Danon disease presents at an earlier age and is classically characterized by skeletal myopathy, cardiomyopathy, and intellectual disability.

**Figure 4:**
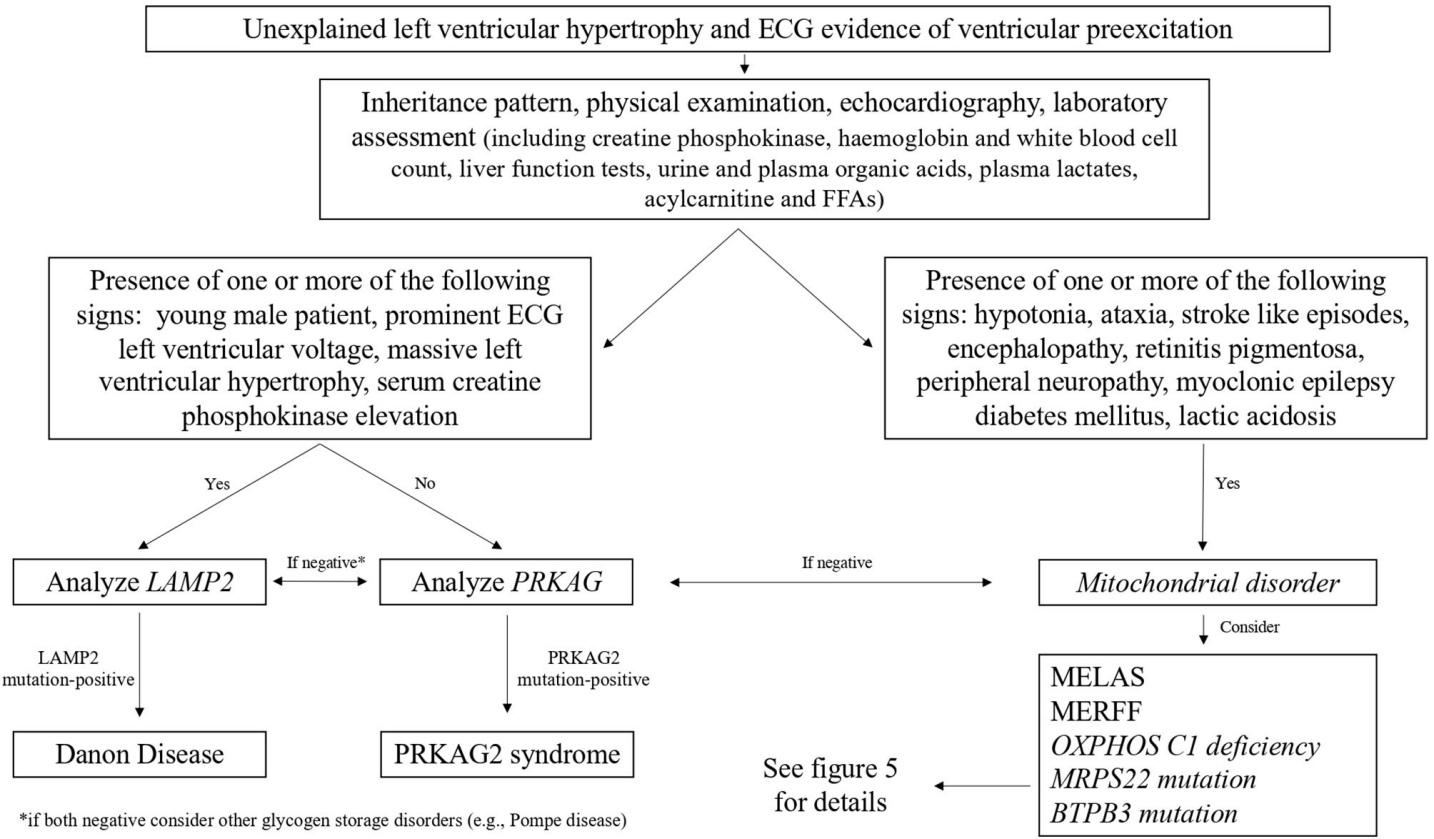
Diagnostic flowchart in patients with unexplained left ventricular hypertrophy and electrocardiographic evidence of ventricular preexcitation. FFA, free fatty acids; MELAS, mitochondrial encephalomyopathy, lactic acidosis, stroke-like episodes; MERRF, myoclonic epilepsy with ragged red fibers.

Danon cardiomyopathy is progressive and usually shows a severe concentric LV hypertrophy (92), which can later progress to LV dilation and heart failure in about 10% of males (93). Conduction abnormalities are also present, manifesting nearly in all of the affected males, and the most common electrocardiographic feature is a short PR interval (preexcitation), present in nearly two thirds of patients (93); the diagnostic flowchart of patients with unexplained left ventricular hypertrophy (LVH) and ventricular preexcitation is shown in Figure 4. Skeletal myopathy manifests in 80–90% of males with progressive proximal muscle weakness (93, 94); it is associated with elevated serum creatine kinase (CK) levels, the presence of intracytoplasmic vacuoles containing autophagic material and glycogen, and the absence of LAMP2 protein expression at skeletal muscle biopsy (93). Other common manifestations in males with Danon disease are represented by learning disability and cognitive defects (in 70–100%) and retinal involvement (in 69%) (95).

On the contrary, females with Danon disease are generally less severely affected. Myocardial involvement is present in 60–100% of females, with an equal prevalence of HCM and dilated cardiomyopathy (93); in addition, conduction abnormalities are present in almost all patients (93, 94). Muscle weakness is generally mild to asymptomatic in females, with mild elevation or normal serum CK levels (96). Likewise, learning disabilities and cognitive defects are less frequent.

In patients with clinically suspect of Danon disease or unexplained LV hypertrophy, the diagnosis is supported by the presence of normal acid maltase levels on muscle biopsy, the deficiency of LAMP2 protein by immunohistochemistry, the evidence of autophagic vacuole accumulation by microscopy, and the presence of a disease-causing mutation of *LAMP2* (97). Therefore, genetic testing, due to its non-invasive nature and the inclusion of *LAMP2* in HCM genetic testing panels, actually represents the most common tool for identifying Danon disease.

### Prognosis and Risk of Sudden Cardiac Death

Heart failure and arrhythmia constitute the leading cause of morbidity and mortality in patients with Danon disease. Recently, data from a multicenter European registry have shown that Danon disease runs a malignant course in both genders, due to cardiac complications (98). In particular, among the 57 patients evaluated (30 males and 27 females), 53% had a heart failure hospitalization, 27% underwent heart transplantation or received an LV assist device, and 30% of patients died during follow-up. Moreover, in this cohort, SCD occurred in three female patients, and appropriate implantable cardioverter-defibrillator (ICD) interventions for ventricular tachycardia/fibrillation were evidenced in six patients, underlying the high risk of SCD in this population (98).

Similar to sarcomeric HCM, SCD in Danon disease is associated with the presence of specific risk factors, including symptomatic arrhythmias, severe hypertrophy, reduced ejection fraction, extended fibrosis on cardiac magnetic resonance (CMR), and family history of SCD (99). However, the association between these risk factors and SCD is based on few studies and with small sample size cohort; thus, the indication for ICD implantation must be individualized on a case-by-case basis.

### Treatment

There is no etiological treatment for Danon disease (Table 3), and the management should be based on a multidisciplinary team-based approach (95). The management of HCM should follow the current guidelines (1, 9), considering that in patients with Danon disease it may present earlier and may progress more rapidly compared with sarcomeric forms of HCM, in particular in males. Therefore, management with inotropic negative or chronotropic negative agents should be used with caution in Danon disease, considering the possible development of systolic LV dysfunction and/or atrioventricular block (92), which represents possible complications of the disorder.

Recently, Manso et al. (100) tested the efficacy of AAV-9-mediated gene therapy delivering the human LAMP2B gene in a mouse model of Danon disease and showed that gene therapy restored protein expression in multiple organs, improved metabolic abnormalities and cardiac function, and increased survival.

### Cori–Forbes Disease (Glycogen Storage Disease Type III)

#### Introduction

Among glycogen storage diseases, GSD type III (OMIM code #232400), also called Cori–Forbes disease, is one of the rare disorders of glycogenolysis associated with the development of LV hypertrophy (101). The incidence of GSD type III is ~1:100,000 in the United States (101). GSD type III is an autosomal recessive disorder caused by *AGL* mutations (102), causing non-functional glycogen debranching leading to the storage of limited dextrin.

### Clinical Presentation and Diagnostic Consideration

Two principal forms have been described: GSD type IIIa, characterized by involvement of the liver and the cardiac and skeletal muscle, and GSD type IIIb, by only liver involvement (102, 103). In infants and in children, the principal features are represented by hepatomegaly, hypoglycemia, failure to thrive, recurrent illness, and/or infection (104). Cardiac involvement is common in GSD type IIIa, mostly starting in the first decade of life (104). The International Study on Glycogen Storage Disease (ISGSDIII) (104) showed that cardiac involvement was present in 58% of patients and that nearly two thirds of these patients had electrocardiographic and/or echocardiography signs of LV hypertrophy, whereas the other one third showed a different form of cardiac hypertrophy (i.e., septal, right ventricular, or biventricular hypertrophy). The presence of severe cardiomyopathy is reported in 15% of patients and represents almost the important cause of death in these patients (104, 105). The diagnosis requires liver and/or muscle biopsy or can be formulated through the identification of a disease-causing homozygotic mutation in *AGL* (106).

### Treatment

Unfortunately, a specific treatment for GSD III is not currently available, and current treatments are based on symptomatic and dietary management to control blood glucose levels (104, 106, 107). However, due to its monogenic nature, it is a suitable candidate for AAV-mediated gene therapy. Recently, Lim et al. (108) showed that in mouse models of GSD III, the AAV-mediated gene therapy blocked the glycogen accumulation in both cardiac and skeletal muscle and improved the muscle functions.

### PRKAG2 Syndrome

#### Introduction

PRKAG2 syndrome is a metabolic disorder, inherited with an autosomal dominant pattern, which is characterized by LV hypertrophy, conduction abnormalities, and ventricular preexcitation, caused by mutations in *PRKAG2* (71, 109). *PRKAG2* mutation is reported in nearly 1% of patients with HCM (72) and in 29% of those with both LV hypertrophy and preexcitation on ECG (110, 111). *PRKAG2* encodes for the y2 regulatory subunit of AMP-activated protein kinase (AMPK) (112). *PRKAG2* mutation leads to structural changes of AMPK (113–115), leading to impaired myocyte glucidic uptake, which results in intracellular glycogen and amylopectin deposition, finally causing storage cardiomyopathy (116). PRKAG2 mutations have also been linked to conduction abnormalities and ventricular preexcitation; however, the underlying mechanism of this association is still unclear (117).

### Clinical Presentation and Diagnostic Consideration

PRKAG2 syndrome may present different severity degree of both the ventricular hypertrophy and arrhythmic manifestations (118). The onset of symptoms commonly occurs within the first three decades of life and arrhythmic symptoms (i.e., palpitations, lipotomies, and syncopal episodes) represent the most common disease presentation (72).

The most common electrocardiographic feature of PRKAG2 syndrome is a short PR interval, found in nearly two thirds of patients (72) (Figure 4); other electrocardiographic abnormalities are represented by the right bundle branch block, different patterns of intraventricular conduction abnormalities, and sinoatrial blocks (119). LVH, evidenced thought echocardiography or CMR, often showed an eccentric distribution with a progressive wall thickness increase in the large part of affected individuals (72). CMR is important in early detecting myocardial involvement. In the early phase of the disease, a focal mid-infero-lateral pattern may be present, and TI values may be reduced, while a diffuse myocardial involvement is generally evidenced in patients with an advanced phase of the disease, and the presence of fibrosis causes T1 value elevation (109).

In the advanced phase, evolution forward LV dilation and dysfunction are possible, with the subsequent development of heart failure (120). Supraventricular tachyarrhythmias were evidenced in more than one third of PRKAG2 syndrome patients, and the large part of these showed an accessory pathway on the electrophysiological study (119, 121, 122). They are mainly represented by atrial fibrillation and flutter; and their complications are represented by stroke and ventricular arrhythmias, sometimes leading to SCD (72, 119, 122). Conduction system dysfunctions, such as advanced atrioventricular blocks, marked sinus bradycardia, and sinus blocks are present in about half of PKAG2 syndrome patients, leading, in a large part of them, to pacemaker implantation (1, 112). The family screening and, when indicated, genetic testing are of great importance for diagnosis.

### Prognosis and Risk of Sudden Cardiac Death

Heart failure development and arrhythmia, and both bradyarrhythmia and tachyarrhythmia, constitute the leading cause of morbidity and mortality in patients with PRKAG2 syndrome. SCD occurs in about 10% of PRKAG2 syndrome patients, mainly as a consequence of advanced atrioventricular block or supraventricular tachycardia degenerated in ventricular fibrillation, or from massive hypertrophy (72, 123, 124). However, current data are not sufficient to clearly define the precise pathophysiologic process leading to SCD.

### Treatment

Given the numerous life-threatening consequences of PRKAG2 syndrome, early identification and management of its complications is mandatory. Actually, no specific guidelines have been formulated for PRKAG2 syndrome. The management of HCM should follow the current guidelines (1, 9). Pacemaker implantation is recommended in patients with symptomatic sinus node dysfunction or atrioventricular block. ICD implantation in primary prevention should be performed after a careful evaluation of the individual risk factors, such as familial history of SCD, arrhythmic syncopal episodes, severe LV hypertrophy, non-sustained ventricular tachycardia, and extended fibrosis on CMR (4, 119).

## Lysosomal Storage Diseases

Lysosomal storage diseases are a heterogeneous group of inherited disorders characterized by the accumulation of undigested or partially digested macromolecules, leading to cellular dysfunction and organomegaly. The forms that most commonly cause HCM are represented by mucopolysaccharidoses (MPSs).

### Mucopolysaccharidoses

#### Introduction

The MPSs (OMIM code #252700) are a heterogeneous group of lysosomal storage disorders caused by the functional deficiency of one of the lysosomal enzymes involved in the catabolism of glycosaminoglycans (GAGs) (125). Individuals with MPSs are affected by progressive deposition of incompletely degraded GAGs within potentially in all organ systems, although the specific distribution depends on the specific disease. Except for MPS type II, which is inherited with an X-linked recessive pattern, all the MPSs are inherited with autosomal recessive pattern (126).

### Clinical Presentation and Diagnostic Consideration

Typical manifestations include skeletal and joint deformities, growth and intellectual disability, central nervous system involvement, respiratory difficulty, gastrointestinal disorders, cardiovascular diseases, and ocular and hearing alterations (127). Cardiovascular involvement is generally present, and it occurs earlier and more frequently in MPS type I, type II, and type VI. Cardiac involvement in MPSs is caused by the massive accumulation of dermatan sulfate especially into valves and great vessels, where this GAG is normally present in a large amount. Its deposition leads to the infiltration of granular cells into valves, myocardial walls, coronary arteries, and conduction system inducing inflammation mediated by Toll-like 4 receptor pathway (128). The principal expression of cardiac involvement is represented by valvular regurgitations and stenosis, mainly involving the left-sided valves, caused by thickening of leaflets or papillary muscles and shortening of subvalvular apparatus. The presence of HCM, endocardial thickening, dilated cardiomyopathy with reduced ejection fraction, and pulmonary hypertension has also been described (129, 130). Typically, LV hypertrophy and diastolic dysfunction occur in the early phases, while LV dilation and systolic dysfunction are common in the final disease stage. Moreover, coronary artery disease and systemic hypertension are common in patients with MPSs and have been associated with the diffuse intimal proliferation from GAG deposition, while electrophysiological anomalies such as atrioventricular blocks are related to fibrosis of the conduction system (128, 131, 132).

The enzyme activity assays on fibroblasts, leucocytes, or serum are the gold standard for a definitive diagnosis. Gene analysis can identify the mutations present; in particular, homozygous mutation in the gene encoding alpha-l-iduronidase (*IDUA*) is diagnostic for MPS type I, in the gene encoding iduronate-2-sulfatase (*IDS*) for MPS type II, and in the gene encoding arysulfatase B (*ARSB*) for MPS type VI (125).

### Prognosis and Risk of Sudden Cardiac Death

Regardless of phenotype, all forms of MPS are associated with increased morbidity and mortality. However, the prognosis of patients with MPS mainly varies according to the type of MPS and the residual level of the deficient enzyme. For example, in MPS I H, the most severe phenotype, patients often die in the first decade of life for infectious, respiratory, or cardiac complications.

Cardiovascular cause of death is mainly caused by advanced heart failure, while SCD or death from coronary occlusion is rare (133, 134).

### Treatment

In MPS patients, cardiac surgery for valve disease is often performed with success, and standard pharmacological therapy for heart failure management is commonly used in current clinical practice (128). Actually, ERT and hematopoietic stem cell transplantation (HSCT) represent the standard of care for most MPS diseases (Table 3). ERT, approved for MPSs I, II, IV, VI, and VII, has improved pulmonary function, walking ability, muscular pain, and organomegaly. This treatment has shown a significant increase in systolic and diastolic functions and an important hypertrophy regression. However, ERT and HSCT are ineffective on poorly vascularized sites such as cardiac valves, corneas, and cartilage (128). The most common adverse events with ERT are IgG antidrug antibody formation and infusion-related hypersensitivity reactions (135–137). HSCT is used in all forms of MPS except MPS III, even if several studies show important benefits of this treatment only in MPS I. Its major adverse effects are graft vs. host disease (GVHD) and graft rejection. Beneficial effects are the significant increase of long-term survival, interruption of pulmonary and cardiac disease progression, especially regarding coronary artery occlusion and ventricular hypertrophy, and the attenuation of some neurocognitive symptoms thanks to its ability to cross the brain–blood barrier (BBB) (138). In contrast to ERT, HSCT is considered a permanent treatment. Recently, the association between ERT and HSCT seems to decrease the likelihood of GVHD and improve therapeutic efficacy (139–141). Among new approaches actually undergoing testing, there is gene therapy. This treatment, based on the direct infusion of viral vectors expressing the functional gene (*in vivo* gene therapy) or on the infusion of modified and transduced cells form recipient patient (*ex vivo* therapy), has the advantage to promote continuous enzyme secretion and to be a permanent therapy (142). Pharmacological chaperone therapy aims to provide the correct folding and the highest stability possible of the mutant proteins, avoiding their deposition and aggregation. These little molecules cross BBB and are not immunogenic, but they can present off-target adverse effects and are not currently approved for MPS disorders (143).

## Mitochondrial Disorders

### Introduction

Mitochondrial disorders represent an extremely heterogeneous group of disorders caused by the dysfunction of the mitochondrial respiratory chain. Several proteins are responsible for the integrity of the mitochondrial structure and function, with the major part encoded by the nuclear DNA (nDNA) and the minor part by the mitochondrial DNA (mtDNA) (144).

For this reason, mitochondrial disorders are a challenging area of genetics (144, 145); indeed, this condition results in different possible inheritance patterns of mitochondrial disorder (i.e., autosomal dominant, autosomal recessive, X-linked, and maternal). Mutations in mtDNA or nDNA genes result in mitochondrial dysfunction, thus resulting in mitochondrial disorders (146). Mitochondrial disorders are typically multiorgan disorders, especially involving those organs with high-energy requirement (147), and it can clinically manifest in the neonatal phase, childhood, or adulthood (148).

Because cardiac muscle is one of the high-energy-demanding tissues, the myocardial involvement (i.e., mitochondrial cardiomyopathy) occurs in about 20–40% of children with mitochondrial disease (147), and it can present as an isolated feature or part of a multiorgan involvement (149). HCM is the most common form of mitochondrial cardiomyopathy; however, other forms of cardiomyopathies are possible.

### Clinical Presentation and Diagnostic Consideration

Several mitochondrial syndromes showed a myocardial involvement as a part of their multiorgan spectrum (149). In these conditions, typically associated with mtDNA mutations, myocardial involvement is characterized by cardiomyopathy and/or conduction abnormalities. In particular, myocardial involvement may be associated with mitochondrial encephalomyopathy, lactic acidosis, and stroke-like episodes (MELAS syndrome) (150); myoclonic epilepsy with ragged red fibers (MERRF syndrome) (151); and neurogenic muscle weakness with sensory neuropathy, ataxia, and pigmentary retinopathy (NARP syndrome) (152). Clinical presentation is extremely variable, with the early onset generally associated with worse outcome and the later onset with mild clinical presentation (63).

**Figure 5:**
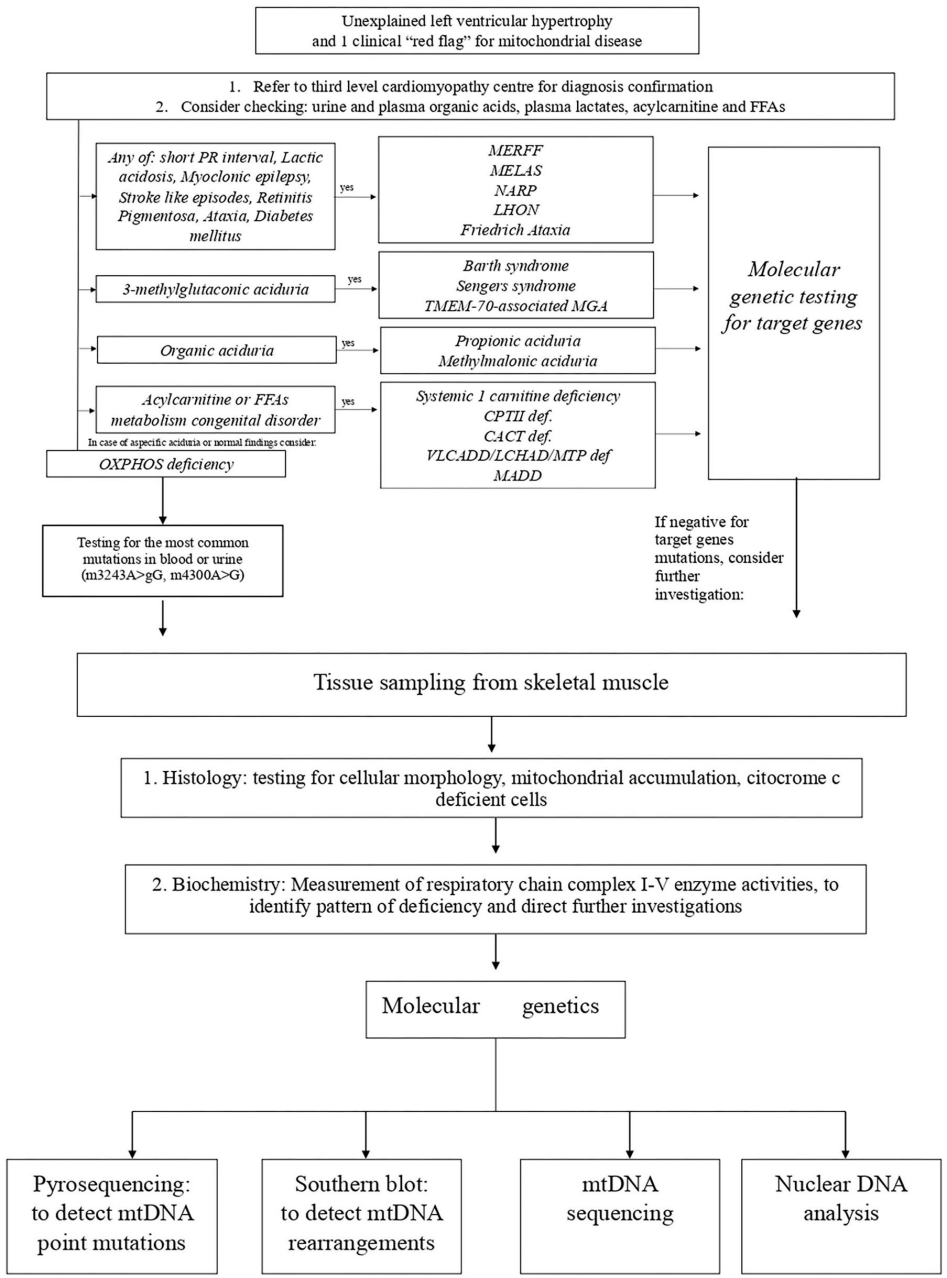
Proposed algorithm for the diagnosis of mitochondrial cardiomyopathies. CACT, carnitine-acylcarnitine translocase; CTPII, carnitine palmitoyltransferase II; FFA, free fatty acids; LCHAD/MTP, long-chain 3-hydroxyacyl-CoA dehydrogenase deficiency; LHON, Leber's hereditary optic neuropathy; MADD, multiple-acyl-CoA dehydrogenase deficiencies; MELAS, mitochondrial encephalomyopathy, lactic acidosis, stroke-like episodes; MERRF, myoclonic epilepsy with ragged red fibers; MGA, methylglutaconic aciduria; NARP, neurogenic muscle weakness with sensory neuropathy; VLCADD, very-long-chain acyl-CoA dehydrogenase deficiency.

Next to the classical mitochondrial syndrome, several other mitochondrial disorders associated with HCM have been identified and can be classified in mitochondrial disorders caused by single or multiple respiratory chain complex deficiencies (153), CoQ10 deficiency (154), mitochondrial transporters deficiency, disorders characterized by 3-methylglutaconic aciduria (e.g., Barth syndrome), disorders of mitochondrial iron metabolism (e.g., Friedreich ataxia), and disorders of mitochondrial β-oxidation and carnitine metabolism (149) (Figure 5).

Deficiency of individual or multiple respiratory chain might result from a mutation in mtDNA or nDNA mitochondrial-related genes, and in both these conditions, it is possible that cardiomyopathy manifests as an isolated feature or in the spectrum of a multisystem disorder (153).

A comprehensive diagnostic workup is required to obtain a final diagnosis (Figure 5). The inheritance pattern, the pattern of organ involvement, the presence of specific findings on clinical and instrumental evaluation, and typical biochemical abnormalities might narrow the differential diagnosis.

### Specific Forms: Barth Syndrome

Barth syndrome (OMIM code #302060) is a rare X-linked genetic disease due to mutation in TAZ gene, which encodes for tafazzin, a phospholipid transacylase that plays an important role in the remodeling of cardiolipin. Cardiomyopathy is present in nearly 70% of Barth syndrome patients and appears in the 1st year of age, usually manifesting as dilated cardiomyopathy or LV non-compaction; HCM appears to be rarer (155, 156). Other clinical manifestations include skeletal myopathy, growth delay, neutropenia, and increased urinary excretion of 3-methylglutaconic acid (3-MGCA) (157).

### Specific Forms: Friedreich Ataxia

Friedreich ataxia (OMIM code #22930) is a neurodegenerative disorder, inherited with an autosomal recessive pattern, caused by the homozygous GAA triplet repeat expansion in the *FXN*, which encodes for the protein frataxin. The first symptoms usually appear in the second decades of life, and the clinical presentation includes progressive ataxia, dysarthria, peripheral neuropathy, and diabetes mellitus (158). Cardiovascular involvement, usually manifesting as unexplained LVH appear during adolescence and is present in nearly two thirds of patients; however, in some cases, the disease can progress to progressive LV dilatation and dysfunction, which can result in heart failure and cardiovascular death.

### Treatment

To date, the only mitochondrial disorders with an etiologic treatment are those caused by CoQ10 deficiency and thiamine-responsive disorders (159) (Table 3). However, target therapies are present for specific mitochondrial diseases. For monogenic disorder, one attractive strategy is AAV gene replacement therapy. For example, in mouse models of Barth syndrome, the AAV-mediated gene therapy rescued neonatal demise, prevented cardiac dysfunction, and reversed established heart disease (160), suggesting that gene therapy might be a therapeutic option for patients with Barth syndrome.

In patients with mitochondrial disorders, heart transplantation may be considered in patients with severe isolated cardiomyopathy or when the eventually extracardiac manifestations are mild and appear non-progressive (161).

## Conclusions

HCM in children represents a large heterogeneous group of disorders. A comprehensive approach, including medical history, physical examination, detailed cardiac imaging, and attention to possible extra-cardiac abnormalities, is required to suspect a specific disorder and to perform a specific diagnosis. The early referral to third-level cardiomyopathy center may be useful in order to start tailor management and potentially initiate targeted genotype-based therapies for these rare conditions.

## Author Contributions

EM, MR, and GL contributed to the conception and design of the work. EM, MR, ML, FDF, RP, FV, and GL drafted the manuscript. All the authors critically revised the manuscript. All the authors gave final approval and agreed to be accountable for all aspects of work ensuring integrity and accuracy. All the authors have read and agreed to the published version of the manuscript.

## Conflict of Interest

The authors declare that the research was conducted in the absence of any commercial or financial relationships that could be construed as a potential conflict of interest.
